# Novel Prion Strain as Cause of Chronic Wasting Disease in a Moose, Finland

**DOI:** 10.3201/eid2902.220882

**Published:** 2023-02

**Authors:** Julianna L. Sun, Sehun Kim, Jenna Crowell, Bailey K. Webster, Emma K. Raisley, Diana C. Lowe, Jifeng Bian, Sirkka-Liisa Korpenfelt, Sylvie L. Benestad, Glenn C. Telling

**Affiliations:** Colorado State University, Fort Collins, Colorado, USA (J.L. Sun, S. Kim, J. Crowell, B.K. Webster, E.K. Raisley, D.C. Lowe, J. Bian, G.C. Telling);; Finnish Food Authority, Helsinki, Finland (S.-L. Korpenfelt);; Norwegian Veterinary Institute, Ås, Norway (S.L. Benestad)

**Keywords:** prions, chronic wasting disease, CWD, moose, gene-targeted, Gt, transgenic, Tg, mice, Finland, Norway, Nordic

## Abstract

Our previous studies using gene-targeted mouse models of chronic wasting disease (CWD) demonstrated that Norway and North America cervids are infected with distinct prion strains that respond differently to naturally occurring amino acid variation at residue 226 of the prion protein. Here we performed transmissions in gene-targeted mice to investigate the properties of prions causing newly emergent CWD in moose in Finland. Although CWD prions from Finland and Norway moose had comparable responses to primary structural differences at residue 226, other distinctive criteria, including transmission kinetics, patterns of neuronal degeneration, and conformational features of prions generated in the brains of diseased mice, demonstrated that the strain properties of Finland moose CWD prions are different from those previously characterized in Norway CWD. Our findings add to a growing body of evidence for a diverse portfolio of emergent strains in Nordic countries that are etiologically distinct from the comparatively consistent strain profile of North America CWD.

Prions are infectious proteins that cause fatal, incurable neurodegenerative diseases of humans and animals, which include Creutzfeldt-Jakob disease (CJD), sheep scrapie, bovine spongiform encephalopathy, and chronic wasting disease (CWD) of cervids. The extraordinary biology and transmissibility of these disorders stems from the protean conformational properties of the prion protein (PrP). Although the secondary structure of host-encoded cellular PrP (PrP^C^) is predominantly α-helical, during disease its relatively underglycosylated infectious counterpart (PrP^Sc^) assembles into amyloid fibrils with parallel, in-register, intermolecular β-sheets (*1*–*7*). The replicative properties of prions stem from the capacity of PrP^Sc^ to template its conformation on PrP^C^ in a cyclical process resulting in exponential accumulation of prion infectivity (*8*–*11*).

Although they lack nucleic acids, prions exhibit heritable strain properties that influence disease outcomes, including the time between infection and disease onset (incubation period), clinical signs, patterns of neuronal degeneration and PrP^Sc^ deposition in the central nervous system (CNS), and the ability to replicate in non-CNS tissues such as the lymphoreticular system and musculature (*12*). Strain properties also influence the capacity of prions from one species to cause disease in a different species (*13*). Heritable strain information appears to be enciphered by distinct PrP^Sc^ conformations that are faithfully propagated during prion replication (*14*,*15*).

The food-chain transmission of bovine spongiform encephalopathy prions that resulted in a variant form of CJD illustrates the unpredictable potential of emergent strains for zoonotic transmission (*13*). Although novel prion diseases and strain variants continue to arise in increasing numbers of animal species, CWD elicits particular concern (*16*). After its initial description in a captive deer facility (*17*), uncontrolled contagious transmission has resulted in growing numbers of CWD-affected cervids in at least 30 US states and 3 Canada provinces (*18*). Inadvertent importation of subclinically diseased animals from North America led to the establishment of CWD in South Korea (*19*–*21*). CWD was also diagnosed in free-ranging Norwegian reindeer in 2016 (*22*), and additional cases were subsequently identified in growing numbers of moose, red deer, and reindeer from Norway, Sweden, and Finland (*23*).

The development and application of genetically modified, CWD-susceptible mice expressing cervid PrP^C^ (CerPrP^C^) has provided insights into multiple aspects of pathogenesis, including the impact of naturally occurring *PRNP* coding sequence variations (*16*). Whereas North America deer and moose encode glutamine (Q) at codon 226 (CerPrP-Q226), North America elk encode glutamate (E) at this position (CerPrP-E226). Early studies in transgenic (Tg) mice suggested a role for this variation in the selection and propagation of CWD strains (*24*–*26*). To precisely assess the effects of this primary structural difference, we created CWD-susceptible gene-targeted (Gt) mice in which the PrP coding sequence was replaced with CerPrP-Q226 or CerPrP-E226 (*27*). Because the resulting mice, referred to as GtQ and GtE mice, express equivalent, physiologically controlled levels of CerPrP^C^ and are otherwise syngeneic, we were able to ascribe distinct disease outcomes in each line to the effects of these amino acids (*27*). Furthermore, in contrast to previous CWD-susceptible Tg mice, Gt mice recapitulated the lymphotropic properties of CWD strains (*27*,*28*). By using Gt mice, we showed that emergent strains causing CWD in Norway reindeer and moose were unrelated to established North America forms of CWD and that they responded differently to variation at residue 226 (*28*). These findings underscored the utility of Gt mice for accurately defining the strain properties of emergent CWD prions (*28*). The goal of this study was to characterize a newly emergent form of CWD in Finland moose.

## Materials and Methods

### Ethics Statement

We performed all animal work in an Association for Assessment for Accreditation of Laboratory Animal Care–accredited facility in accordance with the Guide for the Care and Use of Laboratory Animals (https://www.ncbi.nlm.nih.gov/books/NBK54050). All procedures used in this study were performed in compliance with and were approved by the Colorado State University Institutional Animal Care and Use Committee.

### Isolates

The Finland moose (M-F1) CWD isolate was from a free-ranging female moose identified to be sick and later found dead in 2018. The North America moose (M-US1) CWD isolate was from a captive moose orally inoculated with a pooled preparation of deer CWD. North America elk CWD isolate (E-US1), and Norway moose CWD isolates (M-NO1, M-NO2, and M-NO3) have been described previously (*28*).

### Mouse Models and Incubation Time Assay

Development and characterization of Tg mice expressing cervid PrP with glutamine at residue 226 (TgQ), Tg mice expressing cervid PrP with glutamate at residue 226 (TgE), GtQ and GtE mice (expressing the same thing as Tg mice), and the prion incubation time assay have been described previously (*24*,*27*–*29*). We used equal numbers of male and female mice in inoculation studies. We confirmed clinical diagnoses of prion disease by PrP^Sc^ detection in the CNS and neuropathologic assessments.

### Analysis of PrP^Sc^ by Western Blotting

We performed analysis of PrP^Sc^ by western blotting as described previously (*27*,*28*). Equivalent amounts of protein were treated with 50 μg/mL of proteinase K (PK; Roche, https://www.roche.com) in the presence of 2% sarkosyl for 1 hour at 37°C. We performed sodium dodecyl sulfate–polyacrylamide gel electrophoresis by using precast 12% discontinuous Bis-Tris gels (Bio-Rad Laboratories, Inc., https://www.bio-rad.com). We detected proteins transferred to Immobilon-FL PVDF membranes (Millipore, https://www.emdmillipore.com) with monoclonal antibody (mAb) PRC5 at a dilution of 1:5,000 and mAb PRC1 at a dilution of 1:2,500 (*30*), followed by horseradish peroxidase–conjugated anti-mouse IgG secondary antibody. We developed membranes by using ECL 2 Western Blotting Substrate (ThermoFisher Scientific, https://www.thermofisher.com).

### Conformational Stability Assay

We conducted a conformational stability assay as described previously (*27*,*28*). We detected PrP^Sc^ on membranes with mAb PRC5 (*30*) at a dilution of 1:5,000, followed by horseradish peroxidase–conjugated goat anti-mouse IgG secondary antibody. We developed membranes with ECL 2 Western Blotting Substrate and scanned them with an ImageQuant LAS 4000 digital camera system (GE Healthcare, https://www.gehealthcare.com) and analyzed signals with ImageQuant TL 7.0 software (GE Healthcare).

### Histoblot Analysis

We performed histoblot analysis as described previously (*31*). We detected PrP^Sc^ on membranes by using mAb PRC5 at a dilution of 1:5,000, followed by alkaline phosphatase conjugated goat anti-mouse IgG (Southern Biotech, https://www.southernbiotech.com) at a dilution of 1:5,000. We developed membranes by using 5-bromo-4-chloro-3-indolyl phosphate/nitro blue tetrazolium (Sigma Aldrich, https://www.sigmaaldrich.com) and captured images with a Nikon SMZ1000 microscope (https://www.microscope.healthcare.nikon.com).

### Neuropathologic and Immunohistochemical Analyses

We dissected brains rapidly after euthanizing the animal and immersion fixed tissues in 10% buffered formalin. We embedded tissues in paraffin and mounted 8 μm–thick coronal microtome sections onto positively charged glass slides. We performed immunohistochemical analyses of PrP in brain sections by using mAb D18 as described previously (*28*).

### Neuropathologic Lesion Profiling

We sectioned paraffin-embedded mouse brains coronally to areas corresponding to 5 levels of the brain containing the 9 mouse brain regions of interest. We captured images of hematoxylin and eosin–stained sections by using a Photometrics CoolSNAP digital camera (https://www.photometrics.com) and an Olympus BX51 fluorescence microscope (https://www.olympus-lifescience.com) and analyzed them by using Olympus cellSens imaging software (standard version). We assessed the severity of vacuolar degeneration in cerebral gray matter from 9 regions, as described previously (*32*), by using a team of 4 investigators, who scored the images on scale of 0 to 4, with 4 corresponding to severe vacuolation.

### Statistical Analyses

We performed statistical analyses by using GraphPad Prism 8.0 software (https://www.graphpad.com). We assessed statistical significance between survival curves of inoculated groups by comparing median times of survival of various inoculated groups using the log-rank (Mantel-Cox) test. We assessed statistical significance between denaturation curves of PrP^Sc^ by comparing log half maximal effective concentration values.

## Results

**Figure 1:**
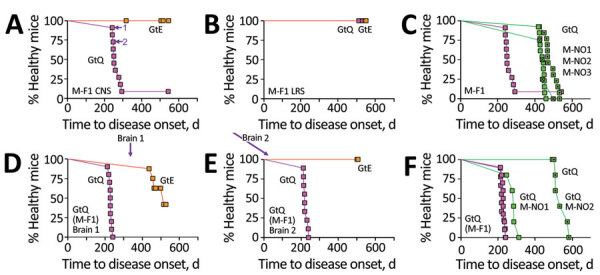
Transmission properties of Finland moose chronic wasting disease (CWD) prions in GtQ and GtE mice. Survival curves of intracerebrally inoculated GtQ and GtE mice are shown. A–C) Primary transmissions; D–F) secondary transmissions. A, B) Transmission to GtE mice (orange squares) and GtQ mice (magenta squares) of CNS homogenate from M-F1 (A) and LRS tissue homogenate from M-F1 (B). Arrows in GtQ mouse brains 1 and 2 (A) used for serial transmissions (D and E). C) Survival of GtQ mice infected with M-F1 from (A) (magenta squares) compared with Norway moose CWD isolates M-NO1 (green squares), M-NO2 (dotted green squares), and M-NO3 (crossed green squares). D) Serial passage of M-F1 prions from GtQ mouse brain 1 to GtE (orange squares) and GtQ (magenta squares). E) Serial passage of M-F1 prions from GtQ mouse brain 2 to GtE and GtQ mice. F) incubation times in GtQ mice of GtQ-passaged M-F1 (brains 1 and 2) from mice in panels D and E (magenta squares) compared with GtQ-passaged M-NO1 (green squares) and M-NO2 (dotted green squares). CNS, central nervous system; GtE, CWD-susceptible gene-targeted mice in which the prion protein coding sequence was replaced with one encoding glutamate at codon 226; GtQ, CWD-susceptible gene-targeted mice in which the prion protein coding sequence was replaced with one encoding glutamine at codon 226; LRS, lymphoreticular system; M-F1, Finland moose 1, M-NO1: Norway moose 1, M-NO2: Norway moose 2, M-NO3: Norway moose 3; p1, primary transmissions; p2, secondary transmissions.

We registered disease after ≈260 days in all but 1 of 11 GtQ mice inoculated with a homogenate of CNS from the index case of Finland CWD, M-F1 (Table 1; Figure 1, panel A). Brain homogenates from 2 diseased GtQ mice, referred to as GtQ (M-F1) brain 1 and brain 2 (Figure 1, panel A), produced disease after ≈225 days following intracerebral inoculation of additional GtQ mice (Table 2; Figure 1, panel D, E). By contrast, all GtE mice inoculated with M-F1 brain homogenate remained free of disease for up to 545 days (Table 1; Figure 1, panel A), and GtQ (M-F1) brain 1 produced disease in only 4 of 7 inoculated GtE mice after ≈470 days (Table 2; Figure 1, panel D), whereas GtQ (MF-1) brain 2 failed to produced disease in GtE mice after >510 days (Table 2; Figure 1, panel E). We conclude that M-F1 prion replication is supported when Q is encoded at residue 226 of host-encoded PrP (CerPrP^C^-Q226) and relatively restricted when E is present at that position. In support of this conclusion, whereas CNS overexpression of CerPrP^C^-Q226 produced faster times to disease in TgQ compared with GtQ mice, only a single inoculated TgE mouse had disease after ≈390 days (Appendix Table 1, Figure 1, panel A).

**Table 1:** Susceptibility of gene-targeted mice to intracerebral challenges with CWD prions from Finland and Norwway moose*

Inoculum	GtQ	GtE
M-F1 CNS	259 +19 (10/11)	>545 (0/9)
M-NO1	440 +13 (13/13)	
M-NO2	486 +34 (13/13)	
M-NO3	451 +33 (4/4)	
M-F1 LRS	>545 (0/10)	>550 (0/6)

*Time to disease onset (incubation time) is expressed as the mean number of days after which inoculated mice first showed ultimately progressive signs of neurologic disease + SD. Parentheses include the numbers of diseased mice/number of inoculated mice. Incubation times of Norway moose CWD prions have been described previously (*27*). GtE, CWD-susceptible gene-targeted mice in which the prion protein coding sequence was replaced with one encoding glutamate at codon 226; GtQ, CWD-susceptible gene-targeted mice in which the prion protein coding sequence was replaced with one encoding glutamine at codon 226; M-F1 CNS, Finland moose 1 (central nervous system); M-F1 LRS, Finland moose 1 (lymphoreticular system); M-NO1, Norway moose 1; M-NO2, Norway moose 2; M-NO3, Norway moose 3.

**Table 2:** Susceptibility of gene-targeted mice to intracerebral challenges with serial passages of Finnish and Norwegian moose CWD prions*

Inoculum	GtQ	GtE
GtQ (M-F1) brain 1	228 +3 (10/10)	>469 +17 (4/7)
GtQ (M-F1) brain 2	223 +4 (9/9)	>507 (0/5)
GtQ (M-NO1)	281 +24 (5/5)	
GtQ (M-NO2)	541 + 5 (5/7)	

*Time to disease onset (incubation time) is expressed as the mean number of days after which inoculated mice first showed ultimately progressive signs of neurologic disease +SD. Parentheses include the numbers of diseased mice/number of inoculated mice. Incubation times of Norway moose CWD prions have been described previously (*27*). CWD, chronic wasting disease; GtE, CWD-susceptible gene-targeted mice in which the prion protein coding sequence was replaced with one encoding glutamate at codon 226; GtQ, CWD-susceptible gene-targeted mice in which the prion protein coding sequence was replaced with one encoding glutamine at codon 226; M-F1, Finland moose 1; M-NO1, Norway moose 1; M-NO2, Norway moose 2.

**Figure 2:**
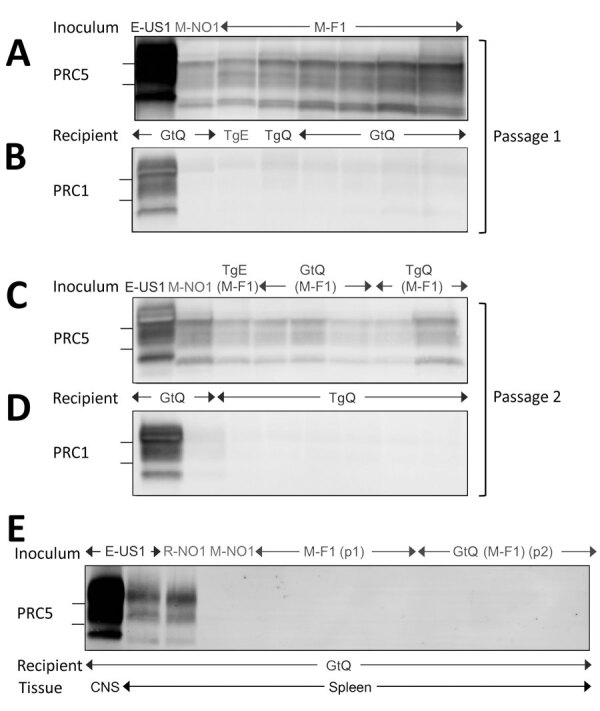
Western blot analyses of cervid prion protein (PrP) scrapie in mice infected with Finland, Norway, and North America chronic wasting disease (CWD) isolates. A, B) Western blots of CNS homogenates from GtQ, TgQ, and TgE mice after primary transmissions E-US1, Norway moose CWD (M-NO1), or Finland moose CWD (M-F1) probed with monoclonal antibody (mAb) PRC5 (A) or PRC1 (B). C–D) Western blots of CNS homogenates of GtQ and TgQ mice infected with E-US1 isolate, Norway isolate M-NO1, and M-F1 passaged through TgE, GtQ, or TgQ, referred to as TgE (M-F1), GtQ (M-F1), and TgQ (M-F1), respectively, probed with mAb PRC5 (C) or PRC1 (D). E) Lane 1, CNS homogenate of GtQ mice infected with E-US1 isolate. Remaining lanes are spleen homogenates from GtQ mice infected with E-US1 isolate, Norway reindeer isolate R-NO1, Norway moose isolate M-NO1, and spleens from infected GtQ mice during primary and secondary transmissions of M-F1. The positions of 25 and 20 kDa molecular weight markers are shown to the left of immunoblots. CNS, central nervous system; E-US1, US elk 1; GtQ, CWD-susceptible gene-targeted mice in which the prion protein coding sequence was replaced with one encoding glutamine at codon 226; M-F1, Finland moose 1; M-NO1, Norway moose 1; PRC1, mAb PCR1; PRC5, mAb PCR5; TgE, transgenic mice expressing cervid PrP with glutamate at residue 226; TgQ, transgenic mice expressing cervid PrP with glutamine at residue 226.

**Figure 3:**
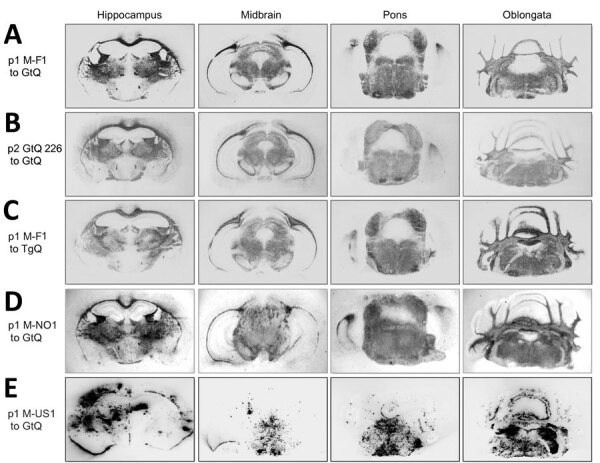
Global central nervous system distribution of cervid prion protein scrapie in Tg and Gt mice infected with Finland, Norway, and North America chronic wasting disease (CWD) cryostat coronal brain sections taken at the level of the hippocampus, midbrain, pons, and oblongata, transferred to slides and then to nitrocellulose. Sections were proteinase K–treated and immunoprobed with monoclonal antibody PRC5 after denaturation. A, B) Passage 1 and 2 of M-F1 in GtQ mice. C) TgQ mice infected with M-F1. D) GtQ mice infected with Norway M-NO1 CWD. E) GtQ mice infected with CWD isolate M-US1. Gt, gene-targeted; GtQ, CWD-susceptible gene-targeted mice in which the prion protein coding sequence was replaced with one encoding glutamine at codon 226; M-F1: Finland moose 1; M-NO1, Norway moose 1; M-US1, US moose 1; p1, primary transmissions; p2, secondary transmissions; Tg, transgenic; TgQ, transgenic mice expressing cervid PrP with glutamine at residue 226.

**Figure 4:**
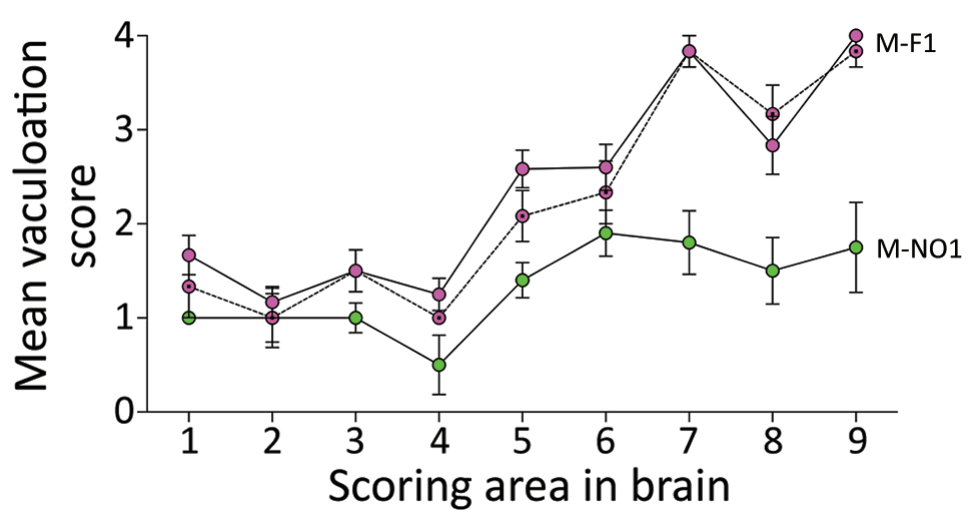
Lesion profiling in GtQ mice infected with Finland and Norway moose chronic wasting disease (CWD) isolates. Lesion profiles in groups of GtQ mice (CWD-susceptible gene-targeted mice in which the prion protein coding sequence was replaced with one encoding glutamine at codon 226) infected with M-F1 (magenta symbols) and Norway moose isolate M-NO1 (green symbols). For M-F1, open circles and solid lines depict primary passage; dotted circles and dashed lines depict second passage. Data points represent the mean +SEM of >5 GtQ mice per group. Brain-scoring areas: medulla (1), cerebellum (2), superior colliculus (3), hypothalamus (4), thalamus (5), hippocampus (6), septum (7), retrosplenial and adjacent motor cortex (8), and cingulate and adjacent motor cortex (9). M-F1, Finland moose 1; M-NO1, Norway moose 1.

**Figure 5:**
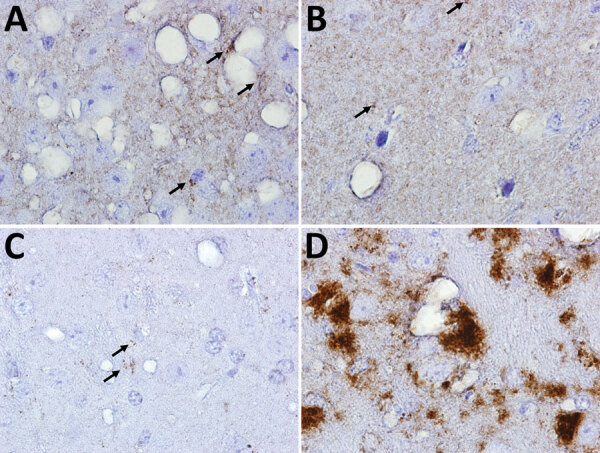
Immunohistochemical analyses of disease-associated prion protein (PrP) in the CNS of transgenic (Tg) and gene-targeted (Gt) mice infected with Finland, Norwway, and North America chronic wasting disease (CWD) isolates. A) GtQ mice (CWD-susceptible Gt mice in which the PrP coding sequence was replaced with one encoding glutamine at codon 226) infected with CWD isolate from Finland moose 1 (MF-1). B) TgE mice (Tg mice expressing cervid PrP with glutamate at residue 226) infected with M-F1. C) GtQ mice infected with CWD isolate from Norway moose 1 (M-NO1). D) GtQ mice infected with CWD isolate from US moose 1. Arrows in panels A, B, and C indicate small puncta of PrP. Immunohistochemistry sections were stained with fragment antigen binding D18. Original magnification ×10.

We confirmed clinical diagnoses of prion disease by using western blotting of PK-resistant CerPrP^Sc^ in brain homogenates (Figure 2, panels A, C), histoblotting of CerPrP^Sc^ in coronal sections (Figure 3, panels A–C), and microscopic analyses of neuronal vacuolation (Figure 4; Appendix Figure 3, panel B) and CerPrP^Sc^ deposition in the CNS (Figure 5, panels A, B). Although spleen homogenates of GtQ mice infected with North America CWD and Norway reindeer CWD prions contained CerPrP^Sc^, there was no evidence of prion replication in the spleens of GtQ mice after primary or secondary passage of the M-F1 isolate, or, as observed previously (*28*), in the spleens of GtQ mice infected with Norway moose CWD isolate M-NO1 (Figure 2, panel E). We conclude that whereas North America and Norway reindeer CWD prions are lymphotropic, Finland and Norway moose CWD prions are nonlymphotropic. In support of this conclusion, neither Gt nor Tg mice had disease after intracerebral inoculation with lymphoid tissue homogenates from M-F1 (Figure 1, panel B; Appendix Figure 1, panel B).

We questioned whether the inefficient transmission of M-F1 prions in GtE and TgE mice could be overcome by adaptation in a CerPrP-E226 background. Serial passage of M-F1 prions from the diseased TgE mouse, referred to as TgE (M-F1) (Figure 2, panel A; Appendix Table 1, Figure 1, panel A), produced disease in GtQ mice after ≈230 days but failed to produce disease in GtE mice after ≈500 days (Appendix Table 2, Figure 1, panel E). Because serial passage of M-F1 prions from diseased TgQ mice also produced disease in GtQ mice after ≈230 days and failed to produce disease in GtE mice after ≈500 days (Appendix Table 2, Figure 1, panel D), we conclude that the M-F1 strain favors conformational conversion of CerPrP^C^-Q226 over CerPrP^C^-E226 regardless of whether prions are composed of CerPrP^Sc^-E226 or CerPrP^Sc^-Q226.

Our previous studies showed that propagation of prions from 3 Norway moose CWD isolates, referred to as M-NO1, M-NO2, and M-NO3, was also supported by expression of CerPrP^C^-Q226 and relatively restricted by CerPrP^C^-E226 (*28*). Despite this shared feature, comparisons of primary transmissions to GtQ mice showed that the mean incubation time of M-F1 was faster than those of M-NO1, M-NO2, or M-NO3 (p<0.0001) (Table 1; Figure 1, panel C). Primary transmission of M-F1 was also faster than Norway moose CWD isolates in TgQ mice (p>0.0001) (Appendix Table 1, Figure 1, panel C). We also observed faster disease onsets when serial transmissions of GtQ (M-F1) brains 1 and 2 were compared with serially transmitted M-NO1 and M-NO2 in GtQ mice (Table 2; Figure 1, panel F) and when M-F1 and Norway moose CWD prions were serially propagated from TgQ to GtQ mice (Appendix Table 2, Figure 1, panel F). Those consistently different disease kinetics during primary and secondary transmissions are indicative of strain differences between Finland and Norway moose CWD prions.

To substantiate that conclusion, we assessed additional strain features in infected GtQ mice. Semiquantitative assessment by brain lesion profiling (*32*) showed that the extent and regional distribution of spongiform degeneration varied depending on whether GtQ mice were infected with Finland or Norway moose CWD prions (Figure 4). Differences between GtQ mice infected with M-F1 and M-NO1 were particularly evident in the septum, retrosplenial and adjacent motor cortex, and cingulate and adjacent motor cortex. In these areas the intensity of spongiform degeneration was more pronounced in GtQ mice infected with M-F1 (Figure 4; Appendix Figure 3, panel A, B). The distinctive lesion profile of M-F1 was sustained after serial passage to additional GtQ mice (Figure 4).

**Figure 6:**
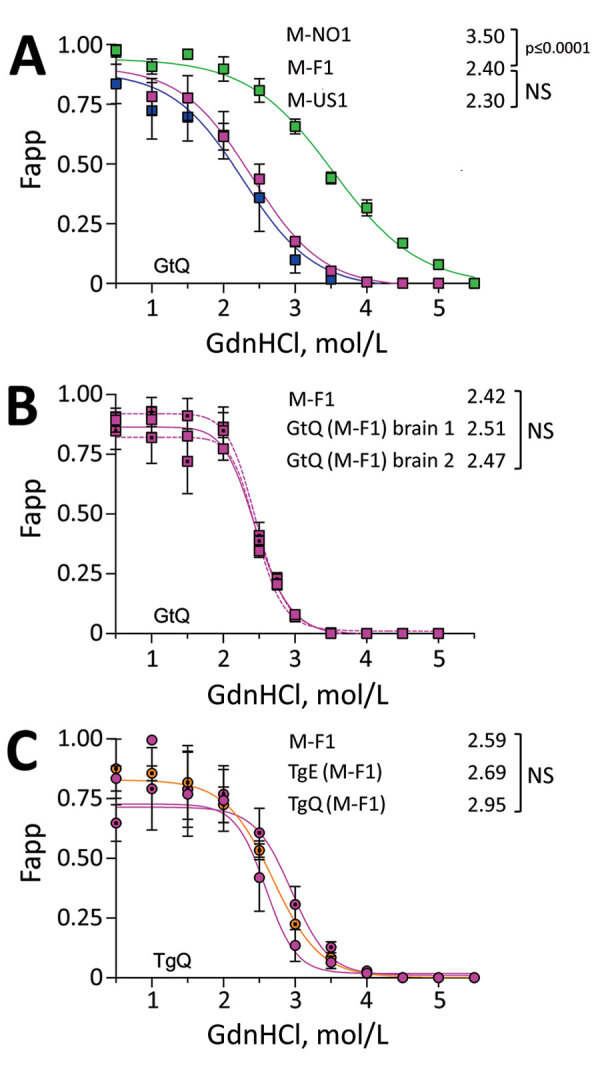
Responses of Finland, Norway, and North America moose chronic wasting disease (CWD) prions propagated in CWD-susceptible mice to increasing concentrations of GdnHCl. A) Responses of CerPrP^Sc^ in central nervous system (CNS) of diseased GtQ mice to proteinase K treatment after denaturation with increasing concentrations of GdnHCl. Magenta symbols indicate M-F1 CWD, green symbols M-NO1 CWD, and blue symbols M-US1 CWD. B) Denaturation profiles of cervid PrP scrapie in CNS of GtQ mice infected with M-F1. Magenta squares and solid lines depict passage 1; dotted magenta squares and dashed lines depict GtQ (M-F1) brains 1 and 2 (passage 2). C) Denaturation profiles of cervid PrP scrapie in the CNS of TgQ mice infected with M-F1 (magenta circles), TgQ mice infected with M-F1 passaged through TgE mice [TgE (M-F1)] (orange circles), and TgQ mice infected with M-F1 passaged through TgQ mice [TgQ (M-F1)] (dotted magenta circles). Proteinase K–resistant PrP^Sc^ was quantified by densitometry of immunoprobed dot blots and plotted against GdnHCl concentration. Sigmoidal dose-response curves were plotted using a 4-parameter algorithm and nonlinear least-square fit. Error bars indicate +SEM of data from analyses of >3 animals per group. GdnHCl_1/2_ values (mol/L) for each infection are reported on the right-hand side of each graph. Significance calculated by pairwise analysis of GdnHCl_1/2_ values from best fit curves. F_app_, fraction of apparent PrP^Sc^ = (maximum signal-individual signal)/(maximum signal-minimum signal); GdnHCl, guanidine hydrochloride; GdnHCl_1/2_, guanidine hydrochloride half-maximal denaturation; GtQ, CWD-susceptible gene-targeted mice in which the prion protein coding sequence was replaced with one encoding glutamine at codon 226; NS, not significant; PK, proteinase K; PrP, prion protein; TgE, transgenic mice expressing cervid PrP with glutamate at residue 226; TgQ, transgenic mice expressing cervid PrP with glutamine at residue 226.

We assessed the responses of CerPrP^Sc^ produced in the brains of mice infected with Finland, Norway, and North America moose CWD prions to denaturation with increasing concentrations of guanidine hydrochloride (GdnHCl) (Figure 6). This measure of PrP^Sc^ stability is associated with conformational variation among prion strains (*33*). Denaturation profiles and concentrations of GdnHCl producing half-maximal denaturation indicated that the stability of CerPrP^Sc^ in GtQ mice infected with M-F1 was equivalent to CerPrP^Sc^ in the brains of GtQ mice infected with North America moose CWD and lower than that of CerPrP^Sc^ in the brains of GtQ mice infected with M-NO1 (p<0.0001) (Figure 6, panel A). The conformational stability of CerPrP^Sc^ produced on primary passage of M-F1 was maintained upon serial transmissions of GtQ (M-F1) brains 1 and 2 to GtQ mice (Figure 6, panel B). We also observed overlapping denaturation curves and comparable half-maximal denaturation values in the range of 2.59 to 2.95 upon infection of TgQ mice with M-F1 prions and M-F1 prions passaged in TgE or TgQ mice (Figure 6, panel C). We conclude that conformational properties of M-F1 prions are distinct from those of M-NO1 prions, they are stable during iterative passages, and they remain unchanged after passage in mice expressing CerPrP^C^-E226.

Despite their different transmission properties, neuropathologic profiles, and conformations, Finland and Norway CWD prions shared certain features. Western blotting showed that levels of CerPrP^Sc^ in the brains of GtQ mice infected with M-F1 and M-NO1 were lower than the levels of CerPrP^Sc^ in the brains of GtQ mice infected with North America CWD (Figure 2). This finding is consistent with previous studies showing consistently reduced CerPrP^Sc^ accumulation after Norway moose CWD prion infection (*28*). The electrophoretic migration profiles of CerPrP^Sc^ resulting from infection of GtQ mice with M-F1 and M-NO1 were both more rapid than CerPrP^Sc^ produced by infection with North America CWD (Figure 2, panel A). This finding also is consistent with our previous descriptions of Norway moose CerPrP^Sc^ (*28*). Finally, also in accordance with previous findings (*28*), CerPrP^Sc^ in the brains of M-F1 and M-NO1 infected mice was relatively refractory to detection by mAb PRC1 compared with CerPrP^Sc^ generated by infection with North America CWD (Figure 2, panel B). The faster migration of CerPrP^Sc^ generated by infection with M-F1 and M-NO1 and its comparative resistance to detection by PRC1 results from PK cleavage downstream from the PRC1 epitope at residue 90 in both strains (Appendix Figure 2). Serial transmissions of M-F1, M-NO1, and North America CWD prions produced CerPrP^Sc^ immunoblotting profiles consistent with primary passages (Figure 2, panel C, D). We speculate that the low levels of PRC1-reactive CerPrP^Sc^ in brain extracts of mice infected with M-F1 and M-NO1 CWD prions (Figure 2, panel B, D) correspond to minor strain components that remain obscured when probing with nondiscriminatory mAbs. Using discriminatory mAbs revealed similar coexistence of multiple PrP^Sc^ types in persons with CJD (*34*).

Analyses of PK-resistant CerPrP^Sc^ in histoblotted coronal brain sections revealed a diffuse, symmetric deposition pattern in GtQ mice infected with M-F1 (Figure 3, panel A) that was indistinguishable from the pattern in GtQ mice infected with M-NO1 CWD (Figure 3, panel D), and consistent with previous analyses of mice infected with Norway moose CWD (*28*). This pattern was recapitulated after iterative passage of GtQ (M-F1) prions to GtQ mice (Figure 3, panel B) and after infection of TgQ mice with M-F1 (Figure 3, panel C). This diffuse pattern differed from the disorganized, asymmetrically distributed amalgamations of CerPrP^Sc^ produced by infection of GtQ mice with North America CWD prions (Figure 3, panel E) (*28*). Microscopic analysis of immunohistochemically stained CNS sections from GtQ mice infected with M-F1 CWD revealed small punctate accumulations of disease-associated CerPrP set against a background of diffuse staining (Figure 5, panel A). We observed a similar pattern in brain sections of TgE mice infected with M-F1 (Figure 5, panel B). We also detected small punctate accumulations in GtQ mice infected with M-NO1 (Figure 5, panel C). By contrast, and consistent with previous findings (*27*,*28*), GtQ mice infected with North America CWD contained intensely staining, amorphous aggregates of disease-associated CerPrP (Figure 5, panel D).

## Discussion

Our previous studies showed that although Gt and Tg mice expressing either CerPrP^C^-E226 or CerPrP^C^-Q226 were susceptible to North America CWD, times to disease onset were more rapid in mice expressing CerPrP^C^-E226 (*24*,*26*–*28*). By comparison, although efficient transmission of Norway moose CWD prions was registered in Gt and Tg mice expressing CerPrP^C^-Q226, their counterparts expressing CerPrP^C^-E226 were resistant to infection or had prolonged incubation times and incomplete attack rates (*28*). Those studies also revealed strain variation among Norway moose CWD isolates, the properties of which were different from Norway reindeer CWD (*28*). The different properties of emergent Norway strains compared with North America CWD made it unlikely that they were causally related. Parallel experiments in bank voles supported this interpretation (*35*). Differences in the strain properties of M-F1 and North America CWD enable us to build on our previous conclusions (*28*,*35*) and to state more broadly that the etiology of Nordic CWD is distinct from North America CWD.

Our analyses reveal that certain characteristics of M-F1 CWD prions overlap with those of Norway moose CWD. M-F1 and Norway moose CWD both propagated more efficiently in mice expressing CerPrP^C^-Q226. Western and histoblot profiles of M-F1 and M-NO1 CerPrP^Sc^ are also indistinguishable. Our findings in this study show that M-F1 prions, like Norway moose CWD prions, are nonlymphotropic. Despite these broadly similar characteristics, other measures, including consistently different disease kinetics, distinct neuropathologic lesion profiles, and variable responses to GdnHCl denaturation, indicate that the strain properties of Finland and Norway moose CWD prions are different. This finding adds to an increasing body of evidence for a surprising variety of strains among Nordic cervids, which stands in contrast to the relatively consistent CWD strain profile among North America deer, elk, and moose. As yet, there appears to be no clear explanation for this diversity of Nordic CWD strains or insights into their origins. Although efficient prion dissemination to peripheral tissues of affected cervids has been cited as a reason for the contagious properties of North America CWD (*36*), the nonlymphotropic properties of Finland and Norway moose CWD strains suggests a decreased potential for contagious propagation. The emergence of increasing numbers of CWD strains in a confined geographic location during a short timeframe presents challenges when considering a sporadic etiology; however, our findings showing dissimilar CWD profiles in Nordic moose can be explained by divergent strains arising from unrelated, stochastic conversion events in different animals. CWD has also been diagnosed in moose in Sweden (*23*). Analyses of the strain properties of those additional emergent CWD cases will be of considerable interest.

Additional findings from this study build on our previous observations and warrant discussion. Seminal studies indicating that related PrP^Sc^ and PrP^C^ primary structures supported optimal prion propagation laid the foundation for the development of Tg mice with susceptibility to human and animal prions (*37*–*39*). Because moose express CerPrP^C^-Q226, we reasoned that the relatively inefficient transmission of M-F1 prions in GtE and TgE mice might be overcome by acclimatization in mice expressing CerPrP^C^-E226, resulting in adapted M-F1 prions composed of CerPrP^Sc^-E226. However, our findings showing comparable transmission profiles of TgE (M-F1) and TgQ (M-F1) prions indicate that M-F1 favors conformational conversion of CerPrP^C^-Q226 over CerPrP^C^-E226 regardless of whether prions are composed of CerPrP^Sc^-E226 or CerPrP^Sc^-Q226. Resistance of M-F1 prions to adapt to a host expressing a single amino acid difference is reminiscent of our description of nonadaptive prion amplification (NAPA) (*40*). Those studies showed that prions produced during particular interspecies transmissions are not adapted for sustained propagation in that new host. Instead, prions resulting from NAPA retain atavistic preferences for their species of origin (*40*). In this context, although M-F1 prions are capable of suboptimal propagation by NAPA using CerPrP^C^-E226 as template for replication, the resulting TgE (M-F1) prions are not adapted for further propagation by CerPrP^C^-E226 but instead retain a preference for conversion of CerPrP^C^-Q226.

Appendix Additional information about novel prion strain as cause of chronic wasting disease in a moose, Finland.
