# Supplementary material for: Novel Prion Strain as Cause of Chronic Wasting Disease in a Moose, Finland
Source: Emerg Infect Dis. 2023 Feb;29(2):323–32. doi: 10.3201/eid2902.220882 (PMC9881765; doi:10.3201/eid2902.220882)
Supplement: Appendix — Additional information about novel prion strain as cause of chronic wasting disease in a moose, Finland. [file 22-0882-Techapp-s1.pdf]

Article DOI: https://doi.org/10.3201/eid2902.220882

Novel Prion Strain as Cause of Chronic Wasting Disease in a Moose, Finland

Appendix

Appendix Table 1. Susceptibility of transgenic mice to intracerebral challenges with Finnish and Norwegian moose CWD prions

Inoculum	TgQ	TgE
M-F1 CNS	234 ± 27 (6/8)	393 (1/5)
M-NO1	297 ± 43 (9/9)	
M-NO2	543 ± 78 (4/5)	
M-NO3	344 ± 101 (9/9)	
M-F1 LRS	>550 (0/9)	>597 (0/9)

Appendix Table 2. Susceptibility of transgenic mice to intracerebral challenges with serial passages of Finnish and Norwegian moose CWD prions

Inoculum	TgQ	TgE
TgE (M-F1)	232 ± 3 (10/10)	>498 (0/7)
TgQ (M-F1)	228 ± 3 (10/10)	>507 (0/6)
TgQ (M-NO1)	271 ± 30 (8/8)	
TgQ (M-NO2)	352 ± 25 (8/8)	
TgQ (M-NO3)	368 ± 17 (8/8)	

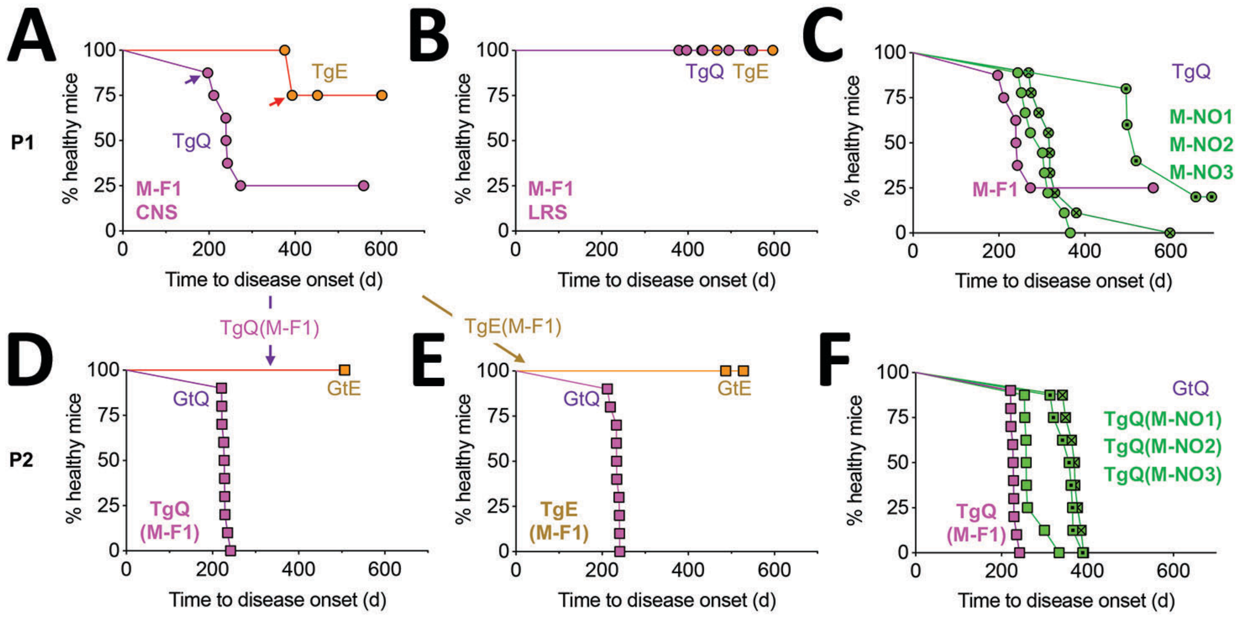

Appendix Figure 1. Transmission properties of Finnish moose CWD prions in TgQ and TgE mice. Survival curves of intracerebrally inoculated TgQ and TgE mice are shown. (A – C), primary transmissions (p1); (D – F), secondary transmissions (p2). Transmission to TgE mice (orange circles) and TgQ mice (magenta circles) of (A) CNS homogenate from M-F1 and (B) lymphoid tissue homogenate from M-F1. Arrows in (A), TgQ and TgE mouse brains used for serial transmissions in D and E. C, incubation times in TgQ mice of TgQ-passaged M-F1 from (A) (magenta circles) compared with Norwegian moose CWD isolates M-NO1 (green circles), M-NO2 (dotted green circles), and M-NO3 (crossed green circles). D, serial passage of TgQ-passaged M-F1 from A to GtE mice (orange squares) and GtQ mice (magenta squares). E, serial passage of TgE-passaged M-F1 from A to GtE and GtQ mice. F, incubation times in GtQ mice of TgQ-passaged M-F1 from (D) (magenta squares) compared with TgQ-passaged M-NO1 (green squares), M-NO2 (dotted green squares), and M-NO3 (crossed green squares). CNS: central nervous system, CWD: chronic wasting disease, Gt: gene-targeted, M-F1: Finnish moose 1, M-NO1: Norwegian moose 1, M-NO2: Norwegian moose 2, M-NO3: Norwegian moose 3, Tg: transgenic.

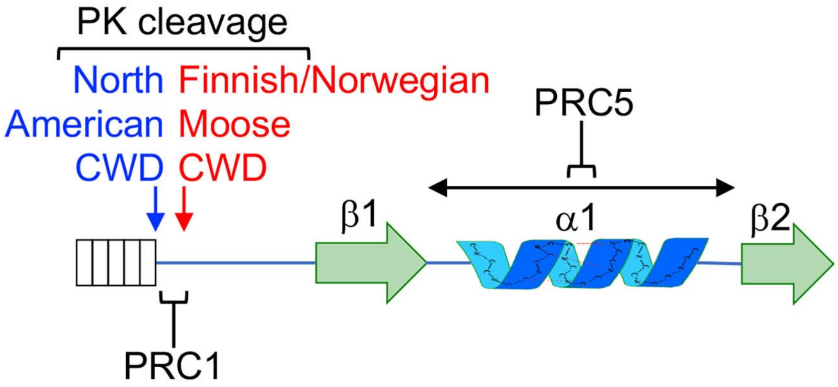

Appendix Figure 2. Epitope map of the cervid prion protein. location of PrP epitopes for mAbs PRC5 and PRC1 and inferred PK cleavage sites for NA and Nordic moose CWD. mAbs: monoclonal antibodies, NA: North American, PK: proteinase K, PrP: prion protein.

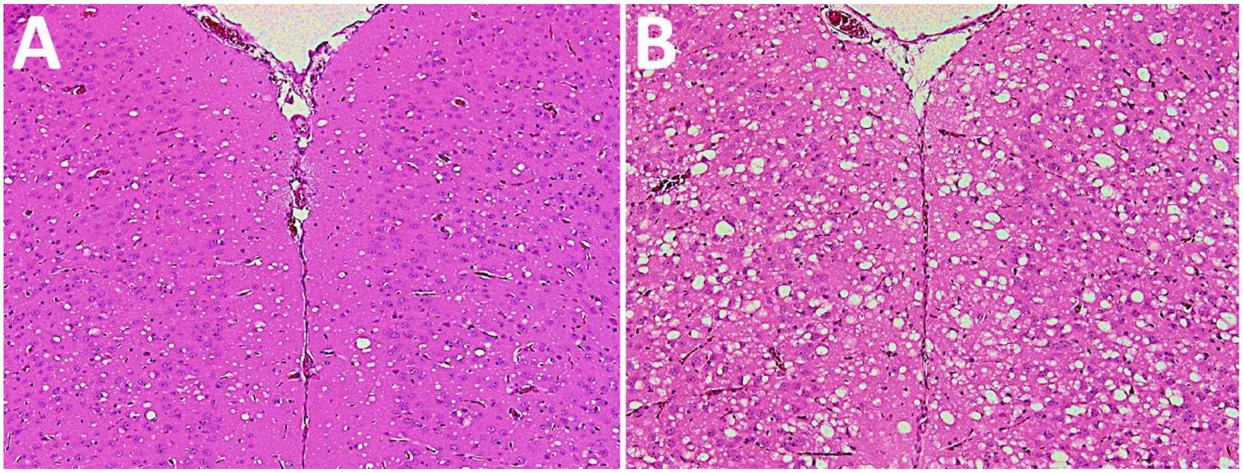

Appendix Figure 3. Neurodegeneration in GtQ mice infected with Finnish and Norwegian moose CWD isolates. Images of hematoxylin and eosin stained sections showing spongiform degeneration in region 9 from (A) M-NO1 infected GtQ and (B) M-F1 infected GtQ mice. Gt: gene-targeted, M-F1: Finnish moose 1, M-NO1: Norwegian moose 1.
